# Ghrelin and ghrelin receptor modulation of psychostimulant action

**DOI:** 10.3389/fnins.2013.00171

**Published:** 2013-09-25

**Authors:** Paul J. Wellman, P. Shane Clifford, Juan A. Rodriguez

**Affiliations:** Behavioral Neuroscience Program, Department of Psychology, Texas A&M UniversityCollege Station, TX, USA

**Keywords:** ghrelin, ghrelin receptors, JMV 2959, locomotion, sensitization, self-administration, feeding

## Abstract

Ghrelin (GHR) is an orexigenic gut peptide that modulates multiple homeostatic functions including gastric emptying, anxiety, stress, memory, feeding, and reinforcement. GHR is known to bind and activate growth-hormone secretagogue receptors (termed GHR-Rs). Of interest to our laboratory has been the assessment of the impact of GHR modulation of the locomotor activation and reward/reinforcement properties of psychostimulants such as cocaine and nicotine. Systemic GHR infusions augment cocaine stimulated locomotion and conditioned place preference (CPP) in rats, as does food restriction (FR) which elevates plasma ghrelin levels. Ghrelin enhancement of psychostimulant function may occur owing to a direct action on mesolimbic dopamine function or may reflect an indirect action of ghrelin on glucocorticoid pathways. Genomic or pharmacological ablation of GHR-Rs attenuates the acute locomotor-enhancing effects of nicotine, cocaine, amphetamine and alcohol and blunts the CPP induced by food, alcohol, amphetamine and cocaine in mice. The stimulant nicotine can induce CPP and like amphetamine and cocaine, repeated administration of nicotine induces locomotor sensitization in rats. Inactivation of ghrelin circuit function in rats by injection of a ghrelin receptor antagonist (e.g., JMV 2959) diminishes the development of nicotine-induced locomotor sensitization. These results suggest a key permissive role for GHR-R activity for the induction of locomotor sensitization to nicotine. Our finding that GHR-R null rats exhibit diminished patterns of responding for intracranial self-stimulation complements an emerging literature implicating central GHR circuits in drug reward/reinforcement. Finally, antagonism of GHR-Rs may represent a smoking cessation modality that not only blocks nicotine-induced reward but that also may limit weight gain after smoking cessation.

## Introduction

The accumulation of excess body fat in obesity is a key etiological factor in current health disorders and early mortality (Olshansky et al., 2005). In spite of efforts to identify the underlying metabolic, hormonal, and behavioral underpinnings of this disorder, there is evidence that the prevalence of obesity is increasing in American children, adults and the elderly (Fakhouri et al., 2012; Flegal et al., 2012; Ogden et al., 2012). In recent years, considerable effort sought to determine effective treatments for obesity including drugs that either alter metabolism, diminish nutrient absorption or suppress appetite (Bray, 2000). One approach has focused on targeting physiological factors that control eating (Bray, 2000) with the hope that strategies might be developed to block orexigenic circuits or to facilitate the activity of circuits that suppress eating. A key example of the latter was the identification of leptin, a peptide that functions to suppress eating. Although there was initial enthusiasm that leptin-modulating drugs might reduce appetite and normalize body weight in obese subjects (Zhang et al., 1994; Nogueiras et al., 2008), leptin treatments are not effective as antiobesity agents (Hukshorn and Saris, 2004). With regard to modulation of orexigenic circuits, a recent candidate peptide has been the gastric peptide ghrelin (GHR), which is the only peripheral orexigenic peptide identified to date (Kojima et al., 1999; Tschop et al., 2000; Ariyasu et al., 2001; Asakawa et al., 2005). Antagonism of ghrelin receptors presumably would suppress appetite and thus represent a novel therapeutic approach for obesity (Asakawa et al., 2003).

## Ghrelin and ghrelin receptors

In 1999, the Kojima group identified GHR as a 28 amino acid peptide secreted from the endocrine cells of the stomach and gut (Kojima et al., 1999). GHR is unique in that this peptide undergoes a posttranslational modification in which an octanoate group is added to the third serine group to form acylated-GHR. (Hosoda et al., 2000; Sakata et al., 2009; Davis et al., 2012). Acyl-GHR (hereafter referred to as GHR) is the endogenous ligand for the growth hormone (GH) secretagogue 1a receptor (GHS-R1a or GHR-R) (Kojima et al., 1999). Unlike GHR, the non-acylated form of ghrelin (des-acyl-ghrelin or DAG) does not activate GHR-Rs (Kojima et al., 1999; Delhanty et al., 2012). Normally, circulating DAG levels are higher than those of GHR levels (Ferrini et al., 2009). Acylation of ghrelin occurs via activation of ghrelin-O-acyl-transferase (GOAT) (Yang et al., 2008) whereas de-acylation of ghrelin can be induced by the enzyme acyl-protein thioesterase 1/lysophospholipase 1 (Satou et al., 2010; Stengel et al., 2011). Diminished GHR signaling would be expected to occur after inhibition of GHR or by activation of de-acylation of GHR. Facilitation of GOAT function should enhance GHR signaling.

The ghrelin peptide is widely distributed throughout the gut and the brain. Ghrelin is primarily secreted from the fundus of the stomach and is transported into the brain across the blood brain barrier (BBB) (Wren et al., 2001; Banks et al., 2002, 2008; Diano et al., 2006). Ghrelin is expressed primarily within the arcuate nucleus (ARC), the lateral hypothalamus (LH), the paraventricular hypothalamic nucleus (PVN), portions of cortex, and the dorsal vagal complex (Cowley et al., 2003; Cowley and Grove, 2004; Hou et al., 2006; Ferrini et al., 2009). Ghrelin receptors appear to modulate a diverse set of physiological and behavioral functions. Ghrelin receptors located within the pituitary (Kojima et al., 1999) and the arcuate nucleus of the hypothalamus (Wren et al., 2000; Mano-Otagiri et al., 2010) play a key role in the release of growth hormone. In the hippocampus, ghrelin receptors may promote long term potentiation (Diano et al., 2006; Banks et al., 2008) and enhance memory consolidation (Carlini et al., 2002; Hansson et al., 2011). Ghrelin receptors have been localized on neurons within the ventral tegmental area (Guan et al., 1997; Abizaid, 2009), which in turn project via the mesolimbic dopamine (DA) pathway to multiple brain regions including the nucleus accumbens (NACc), the amygdala, prefrontal cortex and hippocampus (Fields et al., 2007). Modulation of the mesolimbic dopamine system by ghrelin is likely involved in the capacity of ghrelin to elicit eating and food-related reinforcement (Dickson et al., 2011; Egecioglu et al., 2011; Skibicka and Dickson, 2011) and also plays a key role in the behavioral activating and reward/reinforcement properties of drugs of abuse such as cocaine and nicotine (Jerlhag et al., 2010; Dickson et al., 2011; Wellman et al., 2011, 2012). Finally, ghrelin receptors located on cells of the PVN may play a key role in activation of the hypothalamic-pituitary-adrenal (HPA) axis (Asakawa et al., 2001; Patterson et al., 2010; Cabral et al., 2012), which suggests a role for ghrelin in stress.

Systemic ghrelin can rapidly exert changes in neuronal signaling within the brain. Abizaid reported that systemic infusion of GHR produced synaptic reorganization of neurons within the VTA within 60 min of exposure. VTA cells exposed to ghrelin expressed more excitatory inputs and diminished inhibitory inputs (Abizaid et al., 2006), an outcome similar to that noted in the arcuate nucleus (Cowley et al., 2003; Cowley and Grove, 2004), Systemic infusion of GHR can induce overflow of dopamine in the NACc on a time scale of minutes (Jerlhag et al., 2006a,b; Quarta et al., 2009). These effects of ghrelin on brain neuron function may occur through multiple pathways. One pathway involves a potential activation of GHR-Rs located on afferent fibers of the vagus (Date et al., 2002; Arnold et al., 2006; De Lartigue et al., 2007; Date, 2012) that in turn project to the brainstem. Another potential pathway involves permeation of systemic GHR across the BBB (Banks et al., 2002, 2008; Diano et al., 2006) in a bi-directional fashion (Banks et al., 2002). There is evidence that nutritional state can modulate ghrelin transport into brain with greater transport evident in fasted animals (Banks et al., 2008).

## Psychostimulants and ghrelin

Psychostimulant drugs generally induce behavioral arousal, suppress eating, activate the reward system and, at high doses, may induce symptoms of psychosis (Leibowitz, 1975; Wise and Bozarth, 1987; Kalivas, 2000; Wellman et al., 2009). These effects are often attributed to the capacity of these drugs to activate dopamine-secreting neurons in brain, including neurons of the mesolimbic and corticolimbic dopamine systems (Woolverton and Kleven, 1988; Rothman and Baumann, 2003; Vezina, 2004; Wise, 2004; Di Chiara and Bassareo, 2007). GHR-Rs are located on neurons within the VTA (Guan et al., 1997; Naleid et al., 2005; Abizaid et al., 2006; Diano et al., 2006; Zigman et al., 2006; Abizaid, 2009). The VTA, in turn sends projections to multiple regions including the NACc, amygdala, and prefrontal cortex (Fields et al., 2007) and is thus positioned so as to modulate reinforcement to addictive drugs and natural reinforcers that act via modulation of brain dopamine circuits (Jerlhag et al., 2006a; Abizaid, 2009; Perello et al., 2010; Dickson et al., 2011). In the subsequent sections, we consider strategies for induction of hyperghrelinemia and hypoghrelinemia in relation to psychostimulant function.

## Strategies for modulation of ghrelin circuits

GHR acts as a neuromodulator at brain synapses—either increasing or decreasing neuron activity. One strategy to assess GHR function is to produce variation in plasma GHR levels. There are endogenous rhythms of GHR secretion and these can be temporally related to behavior and other motivated states. Feeding, for example, is known to covary with the peaks and troughs of GHR secretion (Cummings et al., 2001). A more common means to induce hyperghrelinemia would involve exogenous administration of ghrelin or of drugs that act as GHR-R agonists. Systemic or central injections of ghrelin or of GHR agonists are known to induce eating as well as to activate brain mesolimbic circuits involved in reinforcement (Abizaid et al., 2006; Jerlhag et al., 2007; Jerlhag, 2008; Dickson et al., 2011; Skibicka and Dickson, 2011). However, a potential difficulty in such manipulations of the GHR system is that GHR-Rs have a degree of constitutive activity—that is, GHR-Rs may exhibit significant biological activity in the absence of ghrelin (Holst et al., 2003, 2004; Chollet et al., 2009; Petersen et al., 2009) which may limit the magnitude of changes induced by GHR administration or manipulation of the GHR-R.

An alternative strategy to determine the importance of GHR signaling for reinforcement involves inactivation of either GHR or GHR-Rs. A variety of GHR inactivation strategies have been developed (Allas and Abribat, 2013) including inhibition of the enzyme (GOAT) that creates ghrelin (Yang et al., 2008; Al Massadi et al., 2011; Davis et al., 2012; Allas and Abribat, 2013). One recent study reported that knockout of GOAT function (Davis et al., 2012), reduced ghrelin levels and resulted in diminished intake of a palatable high-fat diet “dessert,” a measure of hedonic eating. Other approaches include immunosuppression of ghrelin (Lu et al., 2009) and RNA silencing (Shrestha et al., 2009). Spiegelmers are enantiomers of natural oligonucleotides that have the capacity to bind a peptide such as ghrelin. Spiegelmers may be resistant to degradation and thus may have a long duration of action (Kobelt et al., 2006). Ghrelin spiegelmers have been used to block the orexigenic action of systemic ghrelin and to block the induction by ghrelin of Fos within the arcuate nucleus (Kobelt et al., 2006; Becskei et al., 2008). No study to date has used this method to assess the role of ghrelin in reinforcement. Genomic ablation of the protein that codes for ghrelin has also been accomplished in mice (Sun et al., 2003, 2008) which would allow for an assessment of ghrelin signaling in reinforcement.

A key method for blocking the activation of GHR-Rs by ghrelin involves the use of drugs that function as GHR-R antagonists (Asakawa et al., 2003; Halem et al., 2005; Salome et al., 2009a; Jerlhag and Engel, 2011; Allas and Abribat, 2013; Moulin et al., 2013). The impetus to develop pharmacological antagonists of GHR-Rs is, in part, due to the linkage of GHR-R activation to the induction of feeding (Nakazato et al., 2001; Depoortere, 2009), with the hope that GHR-R inactivation might represent a therapeutic approach for the treatment of obesity. One such drug is the triazole derivative JMV 2959, a selective competitive GHR-R antagonist (Moulin et al., 2007; Salome et al., 2009a,b). JMV 2959 binds to GHR-Rs with low nanomolar affinity (Salome et al., 2009a). As would be expected of a GHR-R antagonist, systemic administration of JMV 2959 dose-dependently blocked the feeding response induced by the synthetic GHR agonist hexarelin (Moulin et al., 2007). JMV 2959 thus represents an important tool for the study of the role of GHR-Rs in drug abuse and other functions.

Another GHR-R inactivation strategy involves genomic manipulation (gene knockout), primarily in mice (Sun et al., 2003, 2008). GHR-R null mice exhibit diminished behavioral activation and reinforcement/reward in response to cocaine, amphetamine and nicotine (Jerlhag et al., 2010; Abizaid et al., 2011; Jerlhag and Engel, 2011; Wellman et al., 2011). GHR-R ablation has also been accomplished in rats using N-ethyl-N-nitrosourea (ENU)-driven target-selected mutagenesis (Zan et al., 2003; Till et al., 2007). GHR-R null rats do not overeat in response to systemic injection (i.p.: 15 nmol) of GHR and importantly, these rats exhibit diminished induction of locomotor sensitization (relative to WT rats) when injected daily with 10 mg/kg cocaine HCl (Clifford et al., 2012). As discussed below, such rats exhibit diminished reinforcement to low-level electrical stimulation of the brain (Wellman et al., 2012).

In the subsequent sections, we consider a variety of issues relating to the interplay of ghrelin and of ghrelin receptors for the impact of psychostimulant drugs such as cocaine, amphetamine or nicotine on behavioral activation and reward/reinforcement.

## Ghrelin modulates the acute hyperlocomotor effects of cocaine

To examine the potential role of ghrelin in psychostimulant action, our preliminary studies focused on the impact of systemic injections of ghrelin on cocaine hyper-locomotion and cocaine-induced conditioned place preference (CPP). The systemic injection route was chosen primarily because the gut is the major source of body ghrelin. In our first study, we examined the impact of 5 nmol ghrelin on cocaine-induced hyperlocomotion (Wellman et al., 2005). The 5 nmol ghrelin dose was chosen, in part, because this dose induced a significant interoceptive cue similar to that produced by food restriction (FR) (Davidson et al., 2005) and secondly because this dose was noted to induce Fos expression in the hypothalamus, but not in the area postrema (Ruter et al., 2003). Male Sprague–Dawley rats were pretreated at −60 min with either 0 (vehicle) or 5 nmol rat ghrelin (i.p.) and then injected (i.p.) at time 0 with 0, 2.5, 5.0, or 10.0 mg/kg cocaine. Locomotor activity was monitored over a 45-min post-cocaine period. Rats received the same ghrelin dose, but a different cocaine dose (in random order) on each of the four drug trials, with each drug trial separated by at least 2 days. Administration of 5 nmol ghrelin-0 mg/kg cocaine slightly increased locomotion relative to that of 0 nmol ghrelin-0 mg/kg cocaine. Cocaine increased locomotion as a function of dose in the 0 nmol ghrelin group, but the effect of cocaine was even greater when preceded by 5 nmol ghrelin. These results indicate that acute injection of ghrelin, at a feeding-relevant dose, augments the acute effects of cocaine on locomotion in rats. A recent study by Jang and colleagues noted a similar outcomes in that injections of ghrelin directly into the NACc enhanced the hyperlocomotor effect of systemic cocaine (15 mg/kg, i.p.), whereas intra-NACc injections of a ghrelin receptor antagonist prevented this effect (Jang et al., 2013). Although GHR-R mRNA is sparse within the NACc, it is detectable and ghrelin infusions into this region can elicit feeding (Naleid et al., 2005; Skibicka et al., 2011a). These studies strongly suggest that acute ghrelin can facilitate the hyperlocomotor effects of a psychostimulant such as cocaine.

## Ghrelin induces cross-sensitization of hyperlocomotion to cocaine

The capacity for repeated administration of a psychostimulant drug to produce behavioral sensitization (i.e., enhanced hyperlocomotion) has been considered as a useful model for understanding the neural underpinnings of drug addiction (Wise and Leeb, 1993; Miller et al., 1999; Davidson et al., 2002; Schoffelmeer et al., 2002; Steketee and Kalivas, 2011). Given that ghrelin alone can induce CPP (Bell et al., 1997) and that a single ghrelin injection can reorganize the inputs of the VTA (Abizaid et al., 2006), we considered the possibility that elevated levels of ghrelin could induce a form of central sensitization in which such treated rats are more reactive to cocaine (Wellman et al., 2008b). Male Sprague–Dawley rats were pretreated daily for 7 days with 0, 5, or 10 nmol rat ghrelin (i.p.) in their home cage. On the 8th day, rats were transported to a testing room, placed in a locomotion chamber for 15 min, and then injected (i.p.) with either 0, 7.5, or 15 mg/kg cocaine with locomotor activity monitored over a 45 min post-cocaine period. Pretreatment with 5 or 10 nmol ghrelin alone did not significantly increase basal locomotion relative to that of the 0 nmol ghrelin group. Rats pretreated with 5 or 10 nmol ghrelin showed an enhanced locomotor response after treatment with 15 mg/kg cocaine relative to rats treated with 0 nmol ghrelin (Wellman et al., 2008b). These results extend our acute locomotion data and suggest that hyperghrelinemia can result in a form of sensitization that facilitates the behavioral activating effects of cocaine.

## Ghrelin enhances cocaine- and food-induced CPP

CPP is a measure of the hedonic quality of a treatment in which a treatment is paired repeatedly with a non-preferred spatial location (Bardo and Bevins, 2000). After multiple pairings, subjects are noted to spend more time in the initially non-preferred location, an outcome which is taken as an indication of the rewarding property of the treatment. Notably, systemic and central administration of GHR alone can induce CPP (Jerlhag, 2008; Jerlhag et al., 2010) and can enhance the CPP induced by cocaine and by food (Davis et al., 2007; Egecioglu et al., 2010; Perello et al., 2010). In a subsequent study, we determined that systemically administered ghrelin (5 nmol/rat) can enhance the rewarding properties of 0.3125 and 0.625 mg/kg cocaine, as indexed by a CPP procedure (Davis et al., 2007). The observation that systemic ghrelin can facilitate the acquisition of CPP to low doses of cocaine suggests that the facilitatory effect of increased ghrelin level is not limited to locomotion and suggests that the effect is not one of a general non-specific enhancement of locomotion, inasmuch as reward in the CPP paradigm is assessed long after the ghrelin-drug pairing (Bardo and Bevins, 2000; Fields et al., 2007).

The capacity of ghrelin to facilitate reward is not limited to psychostimulant-induced reward. Perello et al. (2010) reported that the rewarding effect of exposure to a high-fat pellet in a specific environment was enhanced by pretreatment with 2 ug/g (~18 nmol) ghrelin, but this effect was absent in mice pretreated with a GHR-R antagonist. These latter results strongly implicate ghrelin signaling in reward processes.

## Ghrelin receptor antagonism attenuates psychostimulant-induced hyperlocomotion and reward

Our early experiments involving ghrelin and psychostimulants focused on systemic administration of ghrelin, in part, because of a lack of a readily available method of GHR-R antagonism. Subsequently, the ghrelin receptor antagonist JMV 2959, was made available to our research group by Drs. Fehrentz and Martinez (Salome et al., 2009a; Moulin et al., 2013). Pharmacological inactivation of GHR-Rs by JMV 2959 has been noted to attenuate or to ablate the acute hyperlocomotor and CPP properties of amphetamine, cocaine, ethanol, and most recently that of nicotine (Jerlhag et al., 2009, 2010, 2011; Jerlhag and Engel, 2011). These findings indicate that ghrelin receptors exert a permissive function for the activation of dopamine circuits by psychostimulant drugs (and other drugs of abuse).

Of specific interest to us was the potential impact of GHR-R antagonism on the development of sensitized hyper-locomotion to psychostimulants such as cocaine or nicotine. As noted earlier, repeated injections of ghrelin *per se* can produce a degree of cross-sensitization to cocaine (Wellman et al., 2008b). Drug sensitization reflects a form of neuronal plasticity in which repeated drug administration leads to long-lasting increases in behavioral activation and dopamine overflow within the NACc and it is thought that these effects can lead to enhanced drug taking and addiction (Robinson and Berridge, 2003; Vezina, 2004, 2007). As such, manipulations that attenuate drug sensitization might be of value for the conceptualization and perhaps treatment of addiction either to block the acquisition of sensitization or to block the expression of an already established drug sensitization. Psychostimulants act via the mesolimbic pathway from the VTA to NACc to induce behavioral activation and there is data to suggest that the induction of behavioral induction of sensitization most likely involves the VTA rather than the NACc (Cador et al., 1995). Given an emerging literature linking GHR-Rs to the activation of the VTA (Guan et al., 1997; Cowley et al., 2003; Abizaid et al., 2006; Zigman et al., 2006; Dickson et al., 2011; Skibicka et al., 2011a), we sought to determine the impact of pharmacological blockade of GHR-Rs using the GHR-R antagonist JMV 2959 (Moulin et al., 2007; Salome et al., 2009b) on the development of locomotor sensitization induced by repeated exposure to nicotine (Wellman et al., 2011) as well as to cocaine (Clifford et al., 2012).

**Figure 1:**
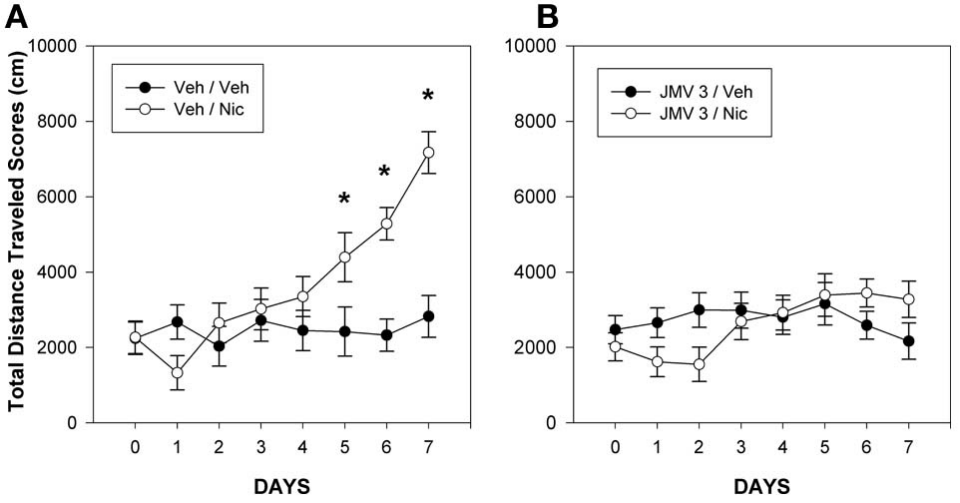
**Mean group total changes in total distance traveled scores (cm/45 min)**. On day 0, the rats were injected with Veh at −5 min prior to the 15 min baseline period and then again with Veh just prior to the 45 min test period. During days 1–7, the rats were injected with either Veh, (panel **A**) or 3 mg/kg JMV 2959 (JMV 3: panel **B**) at −5 min prior to the 15 min baseline period and then injected with either vehicle or 0.4 mg/kg nicotine (Nic) just prior to the 45 min test period on days 1–7. The lines above and below each symbol represent the SEM. Figure reprinted with permission from Regulatory Peptides (Wellman et al., 2011). ^*^p <0.05.

In the nicotine study (Wellman et al., 2011), rats were treated daily for 7 days with 0.4 mg/kg nicotine (s.c), a dose sufficient to result in behavioral sensitization. Half of the rats were pretreated each day with either vehicle or 3 mg/kg JMV 2959. Whereas rats pretreated with vehicle and treated with nicotine developed a robust hyperlocomotor sensitization (Figure 1A), pretreatment with JMV 2959 abolished the development of nicotine sensitization (Figure 1B). The impact of JMV on nicotine sensitization is unlikely to be an artifact of the capacity of JMV 2959 to reduce baseline locomotion. At this dose, there were no baseline differences attributable to JMV 2959 (compare the vehicle-vehicle group in Figure 1A with the JMV 3-vehicle group in Figure 1B). In our cocaine study (Clifford et al., 2012), daily pretreatment with JMV 2959 attenuated the development of locomotor sensitization to 10 mg/kg cocaine, but the magnitude of this effect was less than that noted for nicotine. It should be noted that Abizaid and colleagues have shown that genetic ablation of ghrelin diminished the development of locomotor sensitization of cocaine-induced hyperlocomotion in mice (Abizaid et al., 2011), an outcome which further supports our view that ghrelin and ghrelin receptors may play a permissive role for the induction of psychostimulant-induced locomotor sensitization.

Although the aforementioned findings suggest a permissive role for GHR-Rs for the induction of psychostimulant-induced locomotor sensitization, no study to date has assessed whether antagonism of GHR-Rs would diminish the expression of an already established nicotine locomotor sensitization. In our JMV 2959-nicotine study, JMV 2959 was used throughout the course of nicotine exposure (Wellman et al., 2011). What is needed is an experiment in which nicotine sensitization is produced and then animals are treated with either vehicle or JMV 2959. If JMV 2959 has the capacity to block expression as well as induction of sensitization, this drug may be of value for the treatment of smoking and perhaps other drugs of addiction (Kim et al., 2011).

## Does ghrelin contribute to the capacity of food restriction to enhance psychostimulant action?

FR can increase the rate of acquisition of learned responses for many reinforcers, including food as well as the psychostimulant drugs cocaine or amphetamine (Carroll and Meisch, 1981; Carroll, 1985; Carr, 2002). A link between caloric homeostasis and psychostimulant action in rats is further supported by studies in which FR augments the capacity of psychostimulants to enhance locomotion and to induce CPP (Bell et al., 1997; Zheng et al., 2012). FR also augments the rewarding effects of intracranial self-stimulation of the lateral hypothalamus (LH-ICSS) (Cabeza De Vaca et al., 1998; Fulton et al., 2000); a model system used to explore mechanisms that modulate reinforcement function in brain (Olds and Milner, 1954; Wise, 1996). FR is routinely used by addiction researchers employing intravenous self-administration paradigms to enhance psychostimulant-based reinforcement (Carroll et al., 1979; Carroll and Meisch, 1980, 1981; Carroll, 1985; Comer et al., 1995). Prior studies suggested that signals related to the acute availability of metabolic fuels (e.g., glucose, free fatty acids) are unlikely to wholly account for FR-associated changes in psychostimulant action, inasmuch as short-term glucoprivation or lipoprivation does not alter LH-ICSS in FR rats (Cabeza De Vaca et al., 1998; Fulton et al., 2000; Carr, 2002). Prolonged negative energy balance results in increased expression of neuropeptide Y (NPY) in the hypothalamus; however, administration of NPY does not alter LH-ICSS (Cabeza De Vaca et al., 1998). Thus, the mechanism by which FR enhances psychostimulant effects has remained elusive.

A variety of studies suggest that the orexigenic peptide ghrelin may be the substrate through which FR acts to enhance psychostimulant action. Human plasma ghrelin levels are at a nadir after a meal and then peak prior to the next meal (Cummings et al., 2001). Plasma ghrelin levels increase during periods of FR, and decrease after eating (Toshinai et al., 2001). Importantly, Davidson and colleagues (Davidson et al., 2005) noted that a systemic infusion of ghrelin can generate an interoceptive food cue that resembles that induced by food deprivation (for example, 6 nmol GHR was noted to be comparable to the cue induced by 24 h food deprivation). Collectively, these data suggested that changes in peripheral ghrelin levels occasioned by FR could result in changes in dopamine signaling in brain reinforcement systems that in turn may enhance psychostimulant action. Indeed, our early studies which showed facilitation by ghrelin of acute locomotion to cocaine (Wellman et al., 2005) and enhanced CPP to cocaine (Davis et al., 2007) were generally consistent with the notion that FR may act on a ghrelin-dependent substrate to facilitate psychostimulant action.

To further examine the interaction of FR, ghrelin and psychostimulant function, we examined the capacity of FR to enhance cocaine-induced hyper-locomotion in wild-type (WT) mice vs. GHR-null or GHR-R null mice (Clifford et al., 2011). WT, GHR null, and GHR-R null mice were either restricted to 60% of baseline caloric intake (FR) or allowed to free-feed (FF). Mice were treated with 0, 1.25, 2.5, and 5.0 mg/kg cocaine on separate test days (in random dose order) and forward locomotion was recorded on each drug day for 45 min after drug dosing. FR increased locomotion scores in vehicle treated WT mice, but these increases were diminished in GHR-R and GHR null mice. The doses of cocaine chosen in this study were low (0–5 mg/kg) and did not increase locomotion in FF mice, but produced similar and significant increases in FR mice irrespective of ghrelin status. These preliminary results did not support the contention that GHR pathways are required for the capacity of FR to augment the acute effect of cocaine on locomotion.

Zheng and colleagues conducted psychostimulant testing in the ICSS paradigm during preprandial and postprandial periods in rats fed a single meal per day that represented 40% of their normal daily food intake (Zheng et al., 2013). In such a situation, ghrelin levels are entrained to the feeding period such that ghrelin levels are high during the preprandial period, but low during the postprandial period. Rats received vehicle or amphetamine microinjections into the NACc shell region. All rats were food restricted, but some were tested prior to a daily meal and others tested after that meal. Amphetamine injections shifted the ICSS rate-frequency curve to the left (an outcome indicating enhanced reinforcement) but this effect did not vary by time of feeding. These data suggest that endogenous variation of ghrelin levels of the magnitude noted under these testing conditions was not sufficient to modify psychostimulant reactivity in an ICSS paradigm.

The relatively few studies that have examined the notion that FR may act through a ghrelin-dependent substrate to modify reinforcement are inconclusive. A firm conclusion as to whether ghrelin plays a causal role in enhancing psychostimulant function requires additional study. A key issue is whether conditional GHR-R knockouts would exert an effect on FR activation of psychostimulant locomotion, as opposed to conventional knockouts for which there may be compensations that develop over time. Another issue is the relatively few methods employed to diminish GHR-R function. Additional studies should be conducted using other methods of GHR-R antagonism such as pretreatment with JMV 2959 or de-acylation of GOAT, as specific examples. Additional endpoints (other than locomotion) need to be assessed including CCP as well as intravenous self-administration of cocaine or nicotine. A final issue of concern is the fact that GHR-Rs exhibit a degree of constitutive activity and thus may modulate drug reinforcement, independent of FR and/or ghrelin levels (Holst et al., 2003; Petersen et al., 2009; Steketee and Kalivas, 2011).

## Stress, ghrelin, and food restriction interactions with psychostimulants

Another issue regarding FR and enhancement of psychostimulant function is that ghrelin and GHR-Rs may play an indirect role via activation of the HPA axis. Stressors result in increased CRF secretion from the PVN, secretion of ACTH and enhanced levels of plasma glucocorticoids (GC) such as cortisol (Patterson et al., 2010; Cabral et al., 2012). FR is well-known to enhance circulating levels of ghrelin (Stengel et al., 2011). Interestingly, various forms of psychosocial stress can increase circulating ghrelin levels in rodents (Asakawa et al., 2001; Patterson et al., 2010) as well as humans (Rouach et al., 2007). Moreover, it appears that ghrelin can activate the HPA axis, depending on dose and route of administration. Doses of ghrelin that induce eating (30 nmol) may not alter plasma corticosterone levels when given systemically (Wren et al., 2002). ICV infusion of ghrelin, however, elicits increased plasma ACTH and corticosterone levels (Wren et al., 2002; Stevanovic et al., 2007). Systemic ghrelin (~18 nmol) indirectly activates hypothalamic CRF neurons in mice (Cabral et al., 2012). Another linkage between stress and GHR-R is that GHR-R null mice show diminished HPA response to acute restraint stress (Spencer et al., 2012). In a related vein, stress and enhanced GC signaling has been found to facilitate psychostimulant function. Stress can enhance the magnitude of locomotor activation induced by psychostimulant drugs (Piazza and Le Moal, 1996) and can modify the acquisition (Campbell and Carroll, 2000), maintenance (Carroll, 1985) and reinstatement (De Wit and Stewart, 1981; Carroll, 1985) of self-administration patterns of cocaine in rats (Goeders, 2002, 2003) as does FR (Carroll et al., 1979; Carroll, 1985; Bell et al., 1997). In contrast, blockade of corticosterone signaling can diminish the psychoactive effect of cocaine (Piazza and Le Moal, 1996; Piazza et al., 1996; Marinelli et al., 1997a,b).

These aforementioned studies raise the possibility that FR may act indirectly by increasing GHR signaling which in turn acts at the level of the hypothalamus to enhance the HPA axis modulation of psychostimulant function, perhaps at the level of the VTA (Piazza and Le Moal, 1996; Piazza et al., 1996; Goeders, 2002, 2003). Few studies, however, are available by which to evaluate this hypothesis. One negative finding is that generated by Tessari et al. (2007) in which circulating corticosterone levels were not positively related to the reinstatement of responding for intravenous cocaine in rats. In this same study, plasma levels of ghrelin were related to reinstatement of responding for cocaine. Another negative finding is that acute changes in corticosterone availability does not alter the capacity of FR to sensitize LH-ICSS patterns (Abrahamsen et al., 1995). The same laboratory, however, has generated parallel data in which variation of ghrelin levels does not alter ICSS patterns (Zheng et al., 2013).

Additional experiments are needed to evaluate the potential interaction of stress, GHR signaling and psychostimulant-based reinforcement. A key issue is the need to examine the impact of GHR-R antagonists on self-administration end-points for cocaine and for nicotine. This would include studies of maintenance levels of drug self-administration as well as reinstatement of self-administration (Carroll, 1985; Goeders, 2002, 2003).

## Role of ghrelin receptors in ICSS and drug self-administration

Although psychostimulant-induced locomotor sensitization is thought to contribute to the development of addiction, locomotion is but a proxy for the processes that underlie reinforcement. A number of behavioral paradigms provide models of reinforcement including intracranial self-stimulation (ICSS) (Olds and Milner, 1954; Stein, 1964) and intravenous drug self-administration (IVSA) (Brady and Griffiths, 1976; Carroll and Meisch, 1981; De Wit and Stewart, 1981). In the following sections, we consider published studies in which agonism/antagonism of GHR-Rs alters either ICSS rate-frequency profiles or alters IVSA responding for cocaine and for heroin.

Ablation of the GHR-R in rats has been accomplished using N-ethyl-N-nitrosourea (ENU)-driven target-selected mutagenesis (Zan et al., 2003; Till et al., 2007). GHR-R null rats do not overeat in response to systemic injection (i.p.: 15 nmol) of GHR and importantly, these rats exhibit diminished induction of locomotor sensitization (relative to WT rats) when injected daily with 10 mg/kg cocaine (Clifford et al., 2011). A similar attenuation has been noted in ghrelin null mice (Abizaid et al., 2011). Thus, the lack of GHR or of GHR-Rs compromises the ability of a psychostimulant such as cocaine to induce hyperlocomotor sensitization. To address the issue of whether GHR-null rats exhibit diminished brain reinforcement, we examined the reinforcing action of ICSS in WT and GHR-R null rats. ICSS is a model of brain reinforcement in which animals can press a lever in order to deliver a series of constant-current pulses via an electrode implanted into brain reinforcement circuits (Wise, 1996; Kenny et al., 2003; Carlezon and Chartoff, 2007; Wellman et al., 2008a). Adult male WT and GHR-R null rats were implanted with a single electrode aimed at the LH and then trained, after recovery, to lever-press for ICSS (Wellman et al., 2012). In the rate-frequency procedure, ICSS stimulation intensity is set at a minimum-to-moderate value (75–120 uA) and responding is then recorded as stimulation frequency is reduced in 0.05 log steps from 128 Hz in each minute over 15 min (Carlezon and Chartoff, 2007; Zheng et al., 2013). Responding usually decreases with lower stimulation frequencies. In this study, WT littermates showed normal acquisition of ICSS responding at an intensity of ~70 uA. The lab technician running these rats during acquisition reported that the GHR-R null rats failed to acquire until current intensity was raised 4-fold to 300 uA (see Figure 2 from Wellman et al., 2012). When current intensity was dropped back to 75–100 uA, the GHR-R null did not respond at any stimulation frequency. These results indicate a permissive function for GHR-Rs in ICSS-based reinforcement and generally support the proposition of ghrelin involvement in central reinforcement.

**Figure 2:**
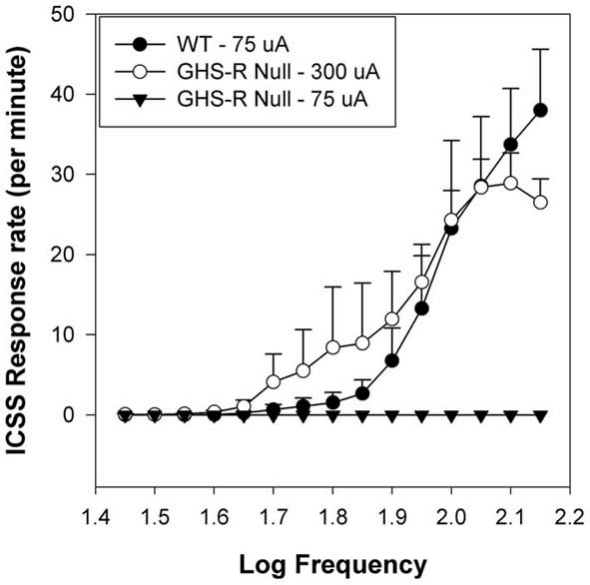
**Mean group Intracranial Self-Stimulation (ICSS) rate-frequency scores for wild-type (WT: *n* = 5) and GHR-R null rats (*n* = 6) as a function of stimulation intensity**. WT rats tested at 75 uA and GHR-R null rats tested at 300 uA exhibit overlapping rate-frequency curves. When GHR-R null rats were tested at 75 uA, their rate-frequency curves dropped to 0. Figure reprinted with permission from Addiction Biology (Wellman et al., 2012).

There are multiple papers that demonstrate that GHR-R inactivation attenuates the CPP induced by cocaine, amphetamine, nicotine, and ethanol (Jerlhag et al., 2009, 2010, 2011; Dickson et al., 2010; Jerlhag and Engel, 2011) as well as by food and by novelty (Egecioglu et al., 2010; Hansson et al., 2012). Few studies, however, have more directly examined the role of GHR-Rs in drug abuse using the IVSA method. In this procedure, rats are trained to lever-press to deliver a drug bolus into their venous system. One variation of the IVSA method involves assessment of the impact of a particular pretreatment on responding for a particular dose of a drug such as cocaine or nicotine (Carroll, 1985). Tessari et al. (2007) reported that circulating GHR levels (but not plasma levels of corticosterone) were positively related to the reinstatement of responding for intravenous cocaine in rats. This study indicates that augmenting GHR levels can facilitate the reinforcing actions of cocaine. An implication of this study is that high plasma ghrelin levels may predispose to relapse in persons formerly addicted to cocaine. A similar effect was noted in a report from Shalev's group in which intracerebroventricular infusions of ghrelin (0.0, 1.5, and 3.0 ug/rat) produced increases in breakpoints on a progressive ratio schedule of heroin reinforcement (Maric et al., 2011).

Another variation of the IVSA method is to examine the potential impact of GHR-R antagonist on reinstatement of previously extinguished drug responding (De Wit and Stewart, 1981; Carroll, 1985). To date, no published study has considered the impact of pretreatment with a GHR-R antagonist on maintenance patterns of responding for cocaine, nicotine or other psychostimulants. With regard to opiate drug self-administration, central administration of the ghrelin receptor antagonist, [D-Lys-3]-GHRP-6 (0.0 or 20.0 ug/rat) had no effect on ongoing heroin self-administration and did not alter reinstatement of heroin responding. To date, this latter work is the only published report to examine the interaction of ghrelin systems and opiate reinforcement. Additional studies of IVSA for cocaine, amphetamine, nicotine and heroin are in order using GHR agonists as well as multiple GHR-R antagonist methods.

## Conclusions

The current literature strongly supports a key role for GHR-Rs within the VTA for the induction of reinforcement for multiple classes of reinforcers including food and perhaps other natural rewards, drugs of abuse and novelty (Jerlhag et al., 2009, 2010; Perello et al., 2010; Dickson et al., 2011; Egecioglu et al., 2011; Jerlhag and Engel, 2011; Skibicka and Dickson, 2011; Hansson et al., 2012; Wellman et al., 2012). The prominent role of GHR-Rs in reinforcement suggests that therapeutic interventions that target these receptors may be of value. Of particular interest for our laboratory is the extent to which GHR-R modulation may offer a valuable route for the treatment of smoking addiction. This disorder remains intractable with few effective therapies available (Laniado-Laborin, 2010). Acute nicotine induces hyper-locomotion, CPP and overflow of DA within the NACc (Jerlhag and Engel, 2011). GHR-Rs are located on cholinergic afferents arising from the laterodorsal tegmental area (LDTg) that project to the VTA, which in turn activate dopamine overflow within the NACc (Jerlhag et al., 2006a). Intra-LDTg infusion of ghrelin enhances locomotion and induces NACc dopamine release (Jerlhag et al., 2007). Within the VTA, GHR-Rs and cholinergic receptors are located on DA neurons that project to the VTA (Dickson et al., 2011: cf. Figure 1, page 82)—thus nicotine may directly alter activity of VTA neurons. In contrast, acute pharmacological blockade of GHR-Rs blocks nicotine-induced locomotion, the induction of CPP by nicotine, and dopamine overflow within the NAcc (Jerlhag and Engel, 2011). Our preliminary behavioral studies (Wellman et al., 2011, 2012) indicate that a drug such as JMV 2959 may offer a route to block nicotine-induced reinforcement, which would be of value for smoking cessation. Additional studies are required to confirm these observations and to demonstrate that GHR-R antagonists can promote smoking abstinence. It is likely that modulation of GHR-Rs may be of use for other drugs of abuse, such as cocaine, alcohol and heroin (Jerlhag et al., 2009, 2010; Maric et al., 2011).

It seems likely that ghrelin acts via multiple circuits to elicit eating (Cowley and Grove, 2004; Naleid et al., 2005; Dickson et al., 2011). A dense concentration of ghrelin receptor protein is localized within the arcuate hypothalamus (Harrold et al., 2008). Ghrelin infusion into the ARC can trigger the release of feeding-relevant peptides and neurotransmitters, which in turn influence food intake and play a role in controlling energy homeostasis (Bagnasco et al., 2003; Cowley et al., 2003; Sato et al., 2005). Ghrelin can alter arcuate nucleus inputs by augmenting NPY signaling, which stimulates food intake, and diminishing proopiomelanocortin (POMC) signaling, which plays a role in the induction of satiety (Cowley et al., 2003). Ghrelin neurons project from the hypothalamus to the brainstem—these fibers likely interact with the cells of the dorsal vagal complex that can also modulate eating (Hou et al., 2006; Hori et al., 2008). In addition to the arcuate nucleus, acute and chronic injections of ghrelin into the VTA as well as the NACc can induce eating (Naleid et al., 2005; King et al., 2011; Skibicka and Dickson, 2011; Skibicka et al., 2011a). Additionally, VTA neurons receive inputs from orexin neurons in the LH, which are also sensitive to ghrelin. What is unknown at the present time is whether these are truly independent circuits or whether these reflect interconnections between these circuits (i.e., involving arcuate or lateral hypothalamic to VTA projections) or interconnections between the NACc and the hypothalamus (Kelley et al., 2005; Dickson et al., 2011). The rewarding effects of palatable food consumption appears to be ghrelin-dependent for both sweets and fats (Egecioglu et al., 2010; Perello et al., 2010; Skibicka and Dickson, 2011; Skibicka et al., 2011b). The involvement of ghrelin for palatable food reward is fascinating since this suggests that antagonism of GHR-Rs may be of value in helping persons to control their overconsumption of palatable foods, which would be of assistance for the treatment of being overweight. Another therapeutic issue for the use of GHR-R antagonists follows from the observation that persons smoke for multiple reasons. Some persons smoke because they enjoy the reinforcement elicited by nicotine but another factor is that smoking can allow people to overeat fat, calories and alcohol (Dallongeville et al., 1998) all while weighing less than normal (Klesges et al., 1989; Zoli and Picciotto, 2012). Cessation of smoking can result in weight gain which can be viewed as unacceptable and therefore diminish treatment compliance (Pomerleau and Saules, 2007). To date, we have few effective interventions by which to limit smoking cessation weight gain (Farley et al., 2012). GHR-R drug antagonists may therefore have a dual utility for the treatment of nicotine addiction: the first being antagonism of the rewarding action of nicotine; the second being obviation of the weight gain often noted following smoking cessation.

### Conflict of interest statement

The authors declare that the research was conducted in the absence of any commercial or financial relationships that could be construed as a potential conflict of interest.
